# Nighttime Light and Thermal Environment Jointly Affect Urban Vegetation Phenology

**DOI:** 10.3390/plants15203094

**Published:** 2026-10-10

**Authors:** Guangliang Jia, Yakai Lei, Jiayi Lin, Pingyang Han, Yuanman Hu, Chunlin Li

**Affiliations:** 1 College of Landscape Architecture, Henan Agricultural University, Zhengzhou 450002, China; jiaguangliang@henau.edu.cn (G.J.); lykfjyl@henau.edu.cn (Y.L.); jiayilin@henau.edu.cn (J.L.); pingyanghan1996@henau.edu.cn (P.H.); 2 CAS Key Laboratory of Forest Ecology and Silviculture, Institute of Applied Ecology Chinese Academy of Sciences, Shenyang 110016, China; huym@iae.ac.cn

**Keywords:** urban vegetation phenology, artificial light at night, thermal environments, urbanization, nonlinear effects

## Abstract

Rapid urbanization has significantly impacted plant growth rhythms through increased surface temperatures and enhanced artificial light at night (ALAN). However, the effects of thermal environments and ALAN on urban vegetation phenology (UVP) remain unclear, limiting our knowledge of how plant growth responds to urbanization and climate change. This study employed high-resolution multi-source data to assess the changing characteristics of the start of the growing season (SOS) and the end of the growing season (EOS) along the urban–rural gradient in Shenyang City. The structural equation model (SEM) and the accumulated local effect model were employed to determine the combined effects and thresholds of thermal environments and ALAN on UVP. We found that the nighttime light duration is associated with delayed autumn vegetation phenology in urban areas. In addition to land surface temperature (standardized path effect, β = 0.41, *p* < 0.05), the nighttime light duration (β = 0.27, *p* < 0.05) and intensity (β = 0.05) were key factors influencing EOS. The impact of nighttime light duration on phenology exhibited distinct seasonal variations, with a significantly greater impact on EOS (β = 0.27, *p* < 0.05) than on SOS (β = 0.14, *p* < 0.05). This might be related to vegetation’s carbon assimilation capacity and its reaction to climate change. These drivers exhibited complex nonlinear relationships with UVP, and the same driver also displayed distinct variation patterns with SOS and EOS. Our findings indicate that nighttime light duration is an important associated factor of plant growth, with great significance for predicting vegetation dynamics under rapid urbanization.

## 1. Introduction

Phenology is the periodic growth rhythm of plants in response to changes in their surrounding environment, including phenomena such as sprouting, leaf unfolding, flowering, and leaf drop [1]. Human-driven rapid urbanization has significantly altered growth activities of urban vegetation and ecosystem dynamics [2]. For instance, the thermal environment and artificial light at night (ALAN) have significantly altered the seasonal rhythms of plant growth [3]. From 2003 to 2020, spring temperatures in 97% of cities were markedly higher than in rural regions, and spring phenology in 66% of cities occurred 6.5 ± 0.9 days earlier than in rural regions [4]. Moreover, ALAN in urban areas grew exponentially from 2014 to 2020, with the onset date of spring phenology in urban vegetation occurring 12.6 days earlier than in rural regions [5]. Changes in thermal environments and ALAN can directly or indirectly alter the timing of spring and autumn phenology in vegetation, thereby influencing vegetation growth mechanisms [6]. Urban vegetation phenology (UVP) directly determines plant distribution and plays a crucial role in related food chain cascading effects, global water and carbon cycles, and responses to future climate change [7]. Therefore, investigating the effects of thermal environments and ALAN on UVP contributes to a better understanding of how urbanization impacts ecosystem dynamics.

Due to rapid urbanization, spring phenology occurs earlier and autumn phenology occurs later in urban areas than in rural areas [8]. UVP is jointly influenced by factors such as temperature, precipitation, and solar radiation. However, current studies primarily attributed changes in UVP to the urban heat island effect yet often overlooked the potential impact of ALAN on vegetation growth [5]. However, controlled experiments have demonstrated that temperature and light jointly regulate plant growth activity [9]. Nighttime light intensity modulates the timing of spring phenological events by adjusting the nonlinear relationship between phenological accumulated temperature requirements and cold-shock requirements [10], while the nighttime light duration alters the circadian rhythm of plants, thereby affecting their growth activities. Especially in urban areas with concentrated ALAN, variations in nighttime light intensity and duration significantly influence the seasonal rhythms of vegetation [11]. Compared to locations without ALAN, ALAN advanced the timing of spring leaf budding by 9 days [12]. Recent studies have demonstrated that increasing the nighttime light intensity can advance the germination period by 4 to 20 days [13]. However, due to the difficulty in measuring the nighttime light duration, studies quantifying its impact on vegetation growth activity are rarely reported [5]. Clarifying the effects of nighttime light intensity and duration on seasonal vegetation rhythms helps to better understand the mechanisms underlying UVP.

The spatial heterogeneity of urban environments causes complex variations in thermal environments and ALAN, resulting in significant uncertainty surrounding their impact on UVP [12]. Previous studies have largely employed a simplistic urban–rural dichotomy, which fails to capture the uneven spatial variation in thermal environments and ALAN across urban–rural gradients [14]. This limitation hinders our understanding of how these factors jointly regulate vegetation phenological changes. Furthermore, most studies have employed traditional statistical analyses to measure the contributions of factors such as ALAN, surface temperature, and climate change to UVP [8]. Structural equation modeling (SEM), as a method for analyzing relationships among complex variables, can simultaneously assess causal relationships among multiple variables [15,16]. Although SEM can intuitively represent path relationships among multiple variables, it struggles to effectively measure the nonlinear changes in driving factors and UVP [7]. Accumulated local effects (ALE) is a machine learning method that rapidly characterizes the overall contribution of influencing factors to the target factor without being constrained by multicollinearity among influencing factors [17]. Combining traditional statistical analysis with machine learning models helps to accurately identify the impact of thermal environments and ALAN on UVP.

Current research has largely focused on the relationship between the urban heat island effect and vegetation growth, often overlooking the combined effects of the thermal environment and nighttime light on vegetation phenology [5,14]. Particularly in high-density urban environments, little attention has been paid to the varying effects of nighttime light intensity and duration on plant growth. As the central city of the Northeast region, Shenyang had reached an urbanization rate of 85.5% by 2024 [18,19]. This provides a good research sample for exploring the effects of thermal environment and ALAN on UVP. This study quantified spatial variations in the start and end of the growing season (SOS and EOS) along an urban–rural gradient using Landsat NDVI data; SEM and ALE were employed to elucidate the effects of thermal environments and ALAN on UVP. The primary objectives of this study were: (1) to elucidate the change characteristics of UVP across urban–rural gradients; (2) to quantify the direct or indirect effects of thermal environments, ALAN, and other factors on UVP; and (3) to identify nonlinear relationships and thresholds between drivers and UVP. Investigating the combined effects of thermal environment and ALAN on UVP contributes to a better understanding of vegetation growth and even ecosystem responses to urbanization.

## 2. Results

### 2.1. Spatial Variations in UVP Along Urban–Rural Gradient

**Figure 1:**
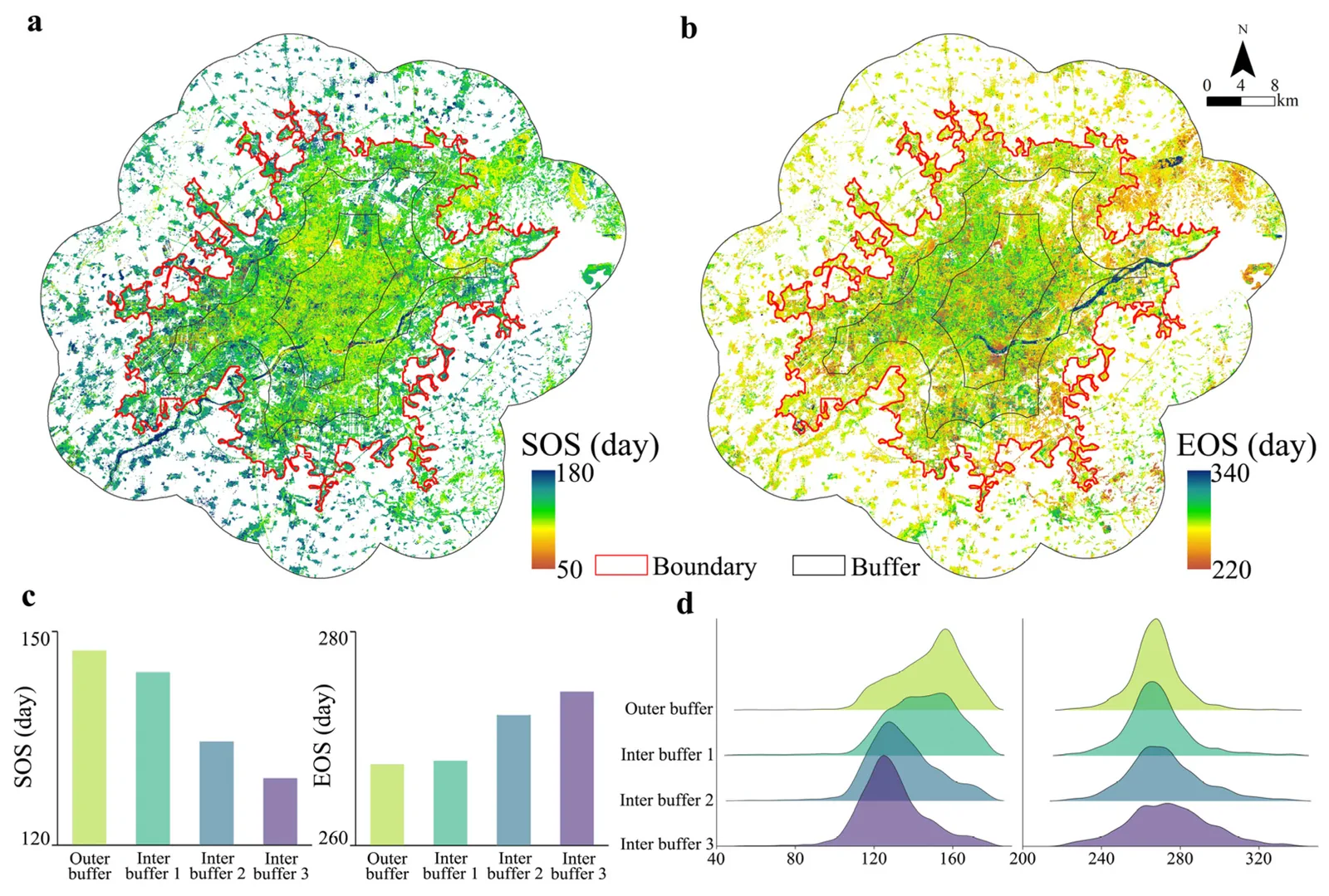
Changes in UVP along the urban–rural gradient. (**a**,**b**) present spatial distribution of SOS and EOS, respectively, and (**c**,**d**) present bar charts and ridge plots of SOS and EOS, respectively. Outer buffer, inter buffer 1, inter buffer 2, and inter buffer 3 represent concentric circular gradients from outer to inner.

Overall, UVP exhibited distinct gradient changes along the urban–rural gradient (Figure 1). Specifically, SOS exhibited a gradually decreasing trend from the outer buffer to the inter buffer 3, with the highest value (147.35 ± 17.26 day of year, DOY) in the outer buffer and the lowest value (129.42 ± 17.10 DOY) in the inter buffer 3. Compared to the outer buffer, SOS in urban areas occurred significantly earlier, with SOS in inter buffer 1, inter buffer 2, and inter buffer 3 advancing by 3.11 days, 12.81 days, and 17.93 days, respectively. In terms of spatial distribution, SOS within urban areas exhibited distinct spatial heterogeneity, with more pronounced SOS advancement occurring in areas near the urban center. In the outer buffer, the SOS in the northeastern mountainous area was markedly higher than in others. As shown in Figure 1c, the SOS value decreased gradually along the urban–rural gradient: outer buffer > inter buffer 1 > inter buffer 2 > inter buffer 3. It was noteworthy that the ridge plots for the outer buffer, inter buffer 2, and inter buffer 3 exhibited a near-normal distribution, whereas inter buffer 1 showed no distinct ridge.

In contrast to the changes in SOS, EOS exhibited a gradually increasing trend from the outer buffer to the inter buffer 3, reaching its lowest value (267.60 ± 13.09 DOY) in the outer buffer and its highest value (274.37 ± 21.22 DOY) in the inter buffer 3. Compared to the outer buffer, the EOS in urban regions exhibited a significant delay trend, with the EOS for inter buffer 1, inter buffer 2, and inter buffer 3 delayed by 0.35 days, 4.61 days, and 6.77 days, respectively. From a spatial distribution perspective, grids with high EOS values were primarily concentrated in inter buffer 2 and inter buffer 3, while grids with low EOS values were concentrated in outer buffer and inter buffer 1. In the outer buffer, the EOS in the northeastern mountainous area was significantly lower than in other areas. As shown in Figure 1c, the EOS value increased gradually along the urban–rural gradient: outer buffer < inter buffer 1 < inter buffer 2 < inter buffer 3. It was worth noting that the ridge plots for EOS and SOS exhibited distinct differences. The ridge plots for EOS showed a normal distribution across the outer buffer, inter buffer 1, inter buffer 2, and inter buffer 3 buffer, with variations primarily observed in ridge heights.

### 2.2. Quantifying the Direct or Indirect Effects of UVP

**Figure 2:**
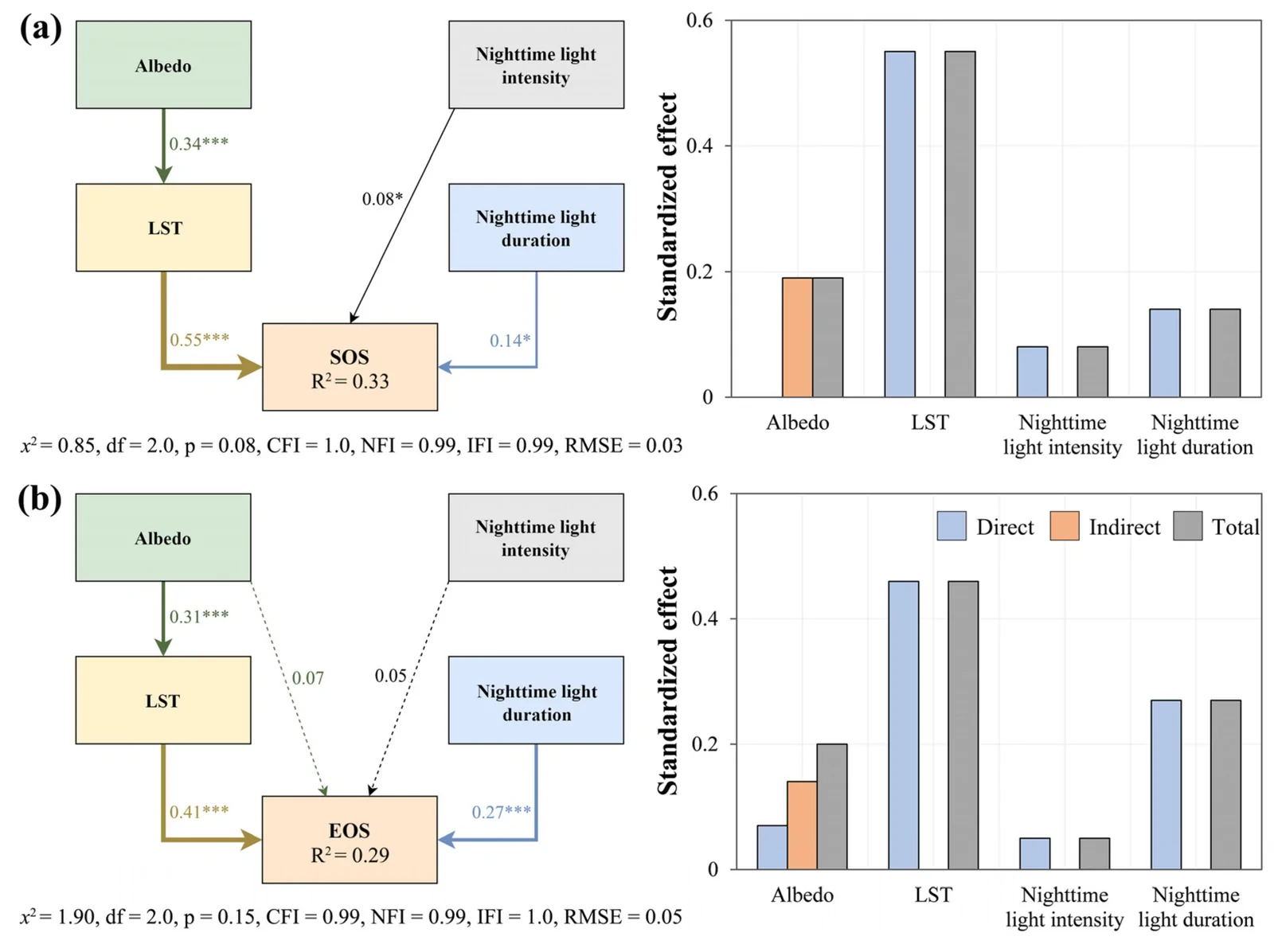
Pathways and path effects of UVP. (**a**,**b**) show the SEM and standardized effects SOS and EOS, respectively. The numerals above the arrows represent standardized path coefficients (* *p* < 0.05, *** *p* < 0.001).

The SEM was applied to measure the direct or indirect effects of drivers on UVP, including thermal environments and ALAN, while comparing the differences in influence across various pathways. The SEM structure, fit statistics, path coefficients, and other parameters are shown in Figure 2. The results demonstrated that the SOS pathway analysis was significant. Land surface temperature (LST), nighttime light intensity, and duration all exhibited positive direct pathway effects on SOS, with LST having the greatest impact (β = 0.55, *p* < 0.05) on SOS. Albedo might indirectly influence SOS by affecting LST. In terms of pathway effects, LST had the greatest impact on SOS, followed by albedo (β = 0.18, *p* < 0.05), nighttime light duration (β = 0.14, *p* < 0.05), and nighttime light intensity (β = 0.08, *p* < 0.05). It was noteworthy that the path effect of nighttime light intensity and duration on SOS is significantly smaller than that of LST.

In the path analysis of EOS, albedo and nighttime light intensity had insignificant effects on EOS, while other factors significantly influenced EOS. LST and nighttime light duration both exhibited positive direct path effects on EOS, with LST having the greatest impact (β = 0.41, *p* < 0.05) on EOS. Among these, albedo not only directly influenced EOS but also indirectly influenced EOS by acting on LST. In terms of pathway effects, LST had the greatest impact on EOS, followed by nighttime light duration (β = 0.27, *p* < 0.05), albedo (β = 0.19, *p* < 0.05), and nighttime light intensity (β = 0.05). The nighttime light duration had a greater impact on both SOS and EOS than the nighttime light intensity. It was noteworthy that the path effect of nighttime light duration exhibits significant seasonal differences, with its impact on EOS being markedly greater than that on SOS.

### 2.3. Identifying the Nonlinear Effects of Drivers on UVP

**Figure 3:**
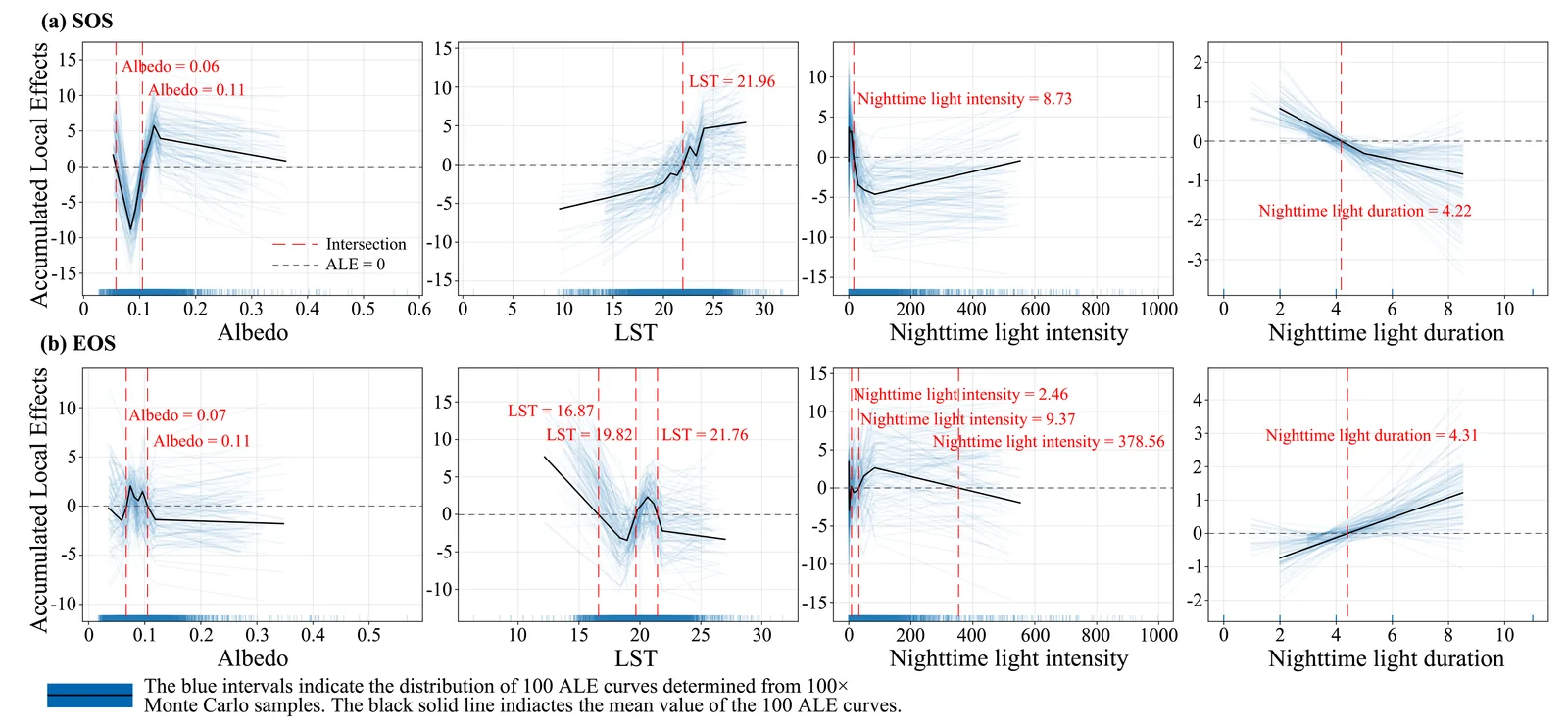
Nonlinear effects of drivers on UVP. (**a**,**b**) represent the effects of albedo, LST, nighttime light intensity, and duration on SOS and EOS, respectively. The intersection point indicates where the curve crosses the line ALE = 0, signifying the value at which the driver’s influence on UVP shifts from positive to negative or from negative to positive.

The ALE was employed to identify the nonlinear effects of drivers on UVP and to compare the change patterns among different drivers. There were complex nonlinear relationships between different drivers and UVP, and the same drivers also exhibited varying change patterns in SOS and EOS (Figure 3). As the albedo increased, the SOS first decreased and then increased, while the EOS first increased and then decreased. As the LST rose, SOS exhibited an overall upward trend, while EOS displayed a complex nonlinear variation. The relationships between these two factors—the nighttime light intensity and duration—and UVP showed significant differences. As the nighttime light intensity increased, SOS first decreased and then increased, while EOS first increased and then decreased. There was a simple monotonic relationship between the nighttime light duration and UVP. As the nighttime light duration increased, the SOS monotonically decreased, while the EOS monotonically increased.

Analyzing the differential impacts of drivers on UVP helped us understand how vegetation growth responds to urbanization processes. Overall, except for LST, the effects of the other factors on SOS and EOS showed opposite changing characteristics. The impacts of albedo on SOS and EOS exhibited opposite changes, while the thresholds for both parameters were nearly consistent. The effects of LST on SOS and EOS showed distinctly different change characteristics. The effect of LST on SOS exhibited a monotonically increasing trend overall, shifting from an inhibitory to a promoting effect at LST = 21.96 °C. The LST promoted EOS when the LST value was below 16.87 °C or between 19.82 °C and 21.76 °C, while it inhibited EOS when the LST value was between 16.87 °C and 19.82 °C or above 21.76 °C. Most nighttime light intensity values fell within the range below 10 nW/cm^2^/sr. When nighttime light intensity was above 8.73 nW/cm^2^/sr, it advanced the SOS, while nighttime light intensity between 9.37 nW/cm^2^/sr and 378.56 nW/cm^2^/sr delayed the EOS. It is noteworthy that nighttime light duration exhibited a monotonic decrease for SOS and a monotonic increase for EOS.

## 3. Discussion

### 3.1. UVP Exhibited Distinct Gradient Changes

Quantifying the variation characteristics of UVP along the urban–rural gradient is helpful to enhance the understanding of how plant growth activities respond to urbanization. We found that within the urban boundary, SOS occurred 11.28 days earlier, while EOS occurred 3.91 days later compared to the outer buffer. This also, to a certain extent, proved that urbanization can effectively cause spring phenology to occur earlier and autumn phenology to occur later [20]. Using concentric circle buffer zones to assess changes in UVP along the urban–rural gradient could minimize the influence of urban scale and land use types on research findings [21]. Along the urban–rural gradient from the outer to the inter buffers, SOS gradually decreased while EOS gradually increased, which is in line with existing research findings [8]. This is primarily because the urban heat island effect is more significantly closer to the urban center, where increased temperatures significantly alter the growth rhythm of plants [14,22]. In the outer buffer, the SOS in the northeastern mountainous area was significantly later than in other areas, while the EOS was significantly earlier than in other areas. This could be attributed to the fact that the northeastern mountainous area is predominantly forested, where phenological changes exhibit significant variations across different land cover types [23]. It is worth noting that the areas around the Hun River, which traverse the urban center, differ significantly from other areas. This might be because these areas are mainly water bodies, and the NDVI values of water bodies are relatively low and stable, and their response to urbanization is not significant.

Currently, only little research has examined the effects of nighttime light intensity on plant growth activities, whereas the effect of nighttime light duration on vegetation phenology has scarcely been reported [24]. Because remote sensing technology struggles to capture the long-term continuous changes in nighttime lights, the assessment of nighttime light duration at medium scales remains rather limited [13]. By combining multi-source remote sensing with field validation, this study comprehensively estimated the spatial pattern of nighttime light duration in Shenyang from the perspective of urban functional zoning. We found that LST increased along the urban–rural gradient, and the nighttime light intensity and duration also increased. However, the variation range in LST was more uniform than that in nighttime lights. This could be attributed to the high density of lighting infrastructure in the urban center, resulting in a markedly uneven increase in nighttime lighting [6]. Nighttime light duration indirectly affects plant growth through mechanisms such as disruption of the photoperiod and thermal effects [25]. Particularly in areas with dense lighting, vegetation phenology is often influenced by the combined effects of the urban heat island and nighttime light. These findings help us to better understand regulation mechanisms of temperature and ALAN that govern vegetation phenological changes in complex urban environments.

### 3.2. Combined Effects of Thermal Environment and ALAN on UVP

In highly heterogeneous urban environments, the LST and ALAN have exhibited significant changes along the urban–rural gradient, making it challenging to assess their individual impacts accurately. Most current research has concentrated on the effects of LST on UVP while neglecting the impact of changes in nighttime light intensity and duration on vegetation growth activities [12]. We employed a systematic approach to determine the combined effects of albedo, LST, nighttime light intensity, and duration on vegetation phenology (Figure 2). The results indicated that the direct effect of LST (β = 0.41, *p* < 0.05) on EOS was significantly higher than that of nighttime light intensity (β = 0.05, *p* < 0.05) and duration (β = 0.27, *p* < 0.05), which was also in line with existing research [13]. Because temperature is the most critical factor influencing plant growth activities, while other factors affect vegetation phenology by altering plants’ temperature sensitivity [7]. Unlike existing research which simply attributed the lengthening of vegetation growing seasons to temperature, our study highlighted that photoperiod (including albedo, nighttime light intensity and duration) also plays a crucial role in altering UVP, particularly in terms of EOS [24]. We found that nighttime light duration (β = 0.27, *p* < 0.05) had a significantly greater impact on EOS than nighttime light intensity (β = 0.05, *p* < 0.05). This might be because sufficient light durations in autumn play a significant role in vegetation senescence [26]. In addition, light wavelength also plays a significant role in regulating plant growth. Future research should focus on the potential effects of different wavelengths, light durations, and light intensities on plant growth.

Clarifying the direct or indirect effects of nighttime light intensity and duration on UVP can help us understand how vegetation growth and even ecosystems respond to urbanization and future climate change. Our findings indicated that, except for LST, albedo, nighttime light intensity, and duration exhibited overall opposite changing characteristics in their effects on SOS and EOS. Compared to factors such as temperature, photoperiod is the most stable “natural signal” through which plants perceive seasonal transitions [27]. When daylight duration reaches a specific threshold in spring, it signals the onset of plant growth, while the shortening of daylight duration in autumn promotes plant senescence and dormancy [28]. Especially in urban environments, the disruption caused by nighttime lights and light pollution makes plant growth rhythms more unpredictable [24]. The effect of LST on SOS exhibited a monotonically changing pattern overall, while the relationship between LST and EOS displayed a complex nonlinear relationship. This might be because low-temperature chilling and accumulated temperature during the pre-season are key factors promoting spring plant growth, while the plant senescence process is subject to complex regulation by multiple environmental factors [29]. For example, during the onset of plant senescence, the shortening of daylight duration serves as the key signal triggering the senescence process, while temperature plays a modulating role in either delaying or accelerating plant senescence [30]. It is worth noting that LST reflects instantaneous daytime surface temperatures and cannot capture nocturnal warming or air temperature changes, making it difficult to effectively characterize the thermal requirements for plant growth. Additionally, the effects of nighttime light duration on SOS and EOS exhibited clear monotonic changes, with their thresholds being relatively close (4.22 and 4.31 h). This is because precise data on nighttime light duration for each pixel was difficult to obtain, resulting in a pronounced linear relationship between nighttime light duration and UVP. However, it is important to note that this mathematical artifact may obscure the true critical thresholds in linear or nonlinear relationships.

### 3.3. Advantages and Limitations

The combined effects of the thermal environment and ALAN on UVP were investigated using SEM, and the nonlinear relationships between UVP and its driving factors were measured using ALE. Current studies have primarily focused on the impact of the urban heat island effect on UVP, with little distinction made between the impacts of thermal environment and ALAN changes on UVP, particularly nighttime light duration [31]. Due to the difficulty in capturing long-term continuous changes in nighttime lights through remote sensing technology, current research on nighttime light duration at the medium scale remains quite limited [12]. This study developed nighttime light duration distribution data in Shenyang by combining multi-source remote sensing with field observations and further distinguished the impacts of nighttime light intensity and duration on vegetation growth activity. Additionally, this study employed ALE to determine the thresholds at which drivers influence UVP, thereby enhancing comprehensive understanding of vegetation growth mechanisms.

Some limitations of this study should be recognized. First, although this study evaluated data on nighttime light duration from the perspective of urban functional zoning, it overlooked the seasonal differences in nighttime light duration. These differences may affect our understanding of real photoperiodic variations and plant physiological mechanisms. Second, to ensure consistency across the overall research data, this study focused solely on phenological changes in a single year. However, phenological changes in a single year are often strongly influenced by climate fluctuations, which might, to some extent, affect the accuracy of the research findings. Furthermore, the extension of plant growing seasons also lengthens the emission period for biogenic volatile organic compounds, thereby exacerbating the formation of ground-level ozone and secondary organic aerosols [32,33]. Future research should focus on the trade-off between the increase in carbon sinks resulting from a longer growing season and the associated ecological costs.

## 4. Materials and Methods

### 4.1. Study Area

**Figure 4:**
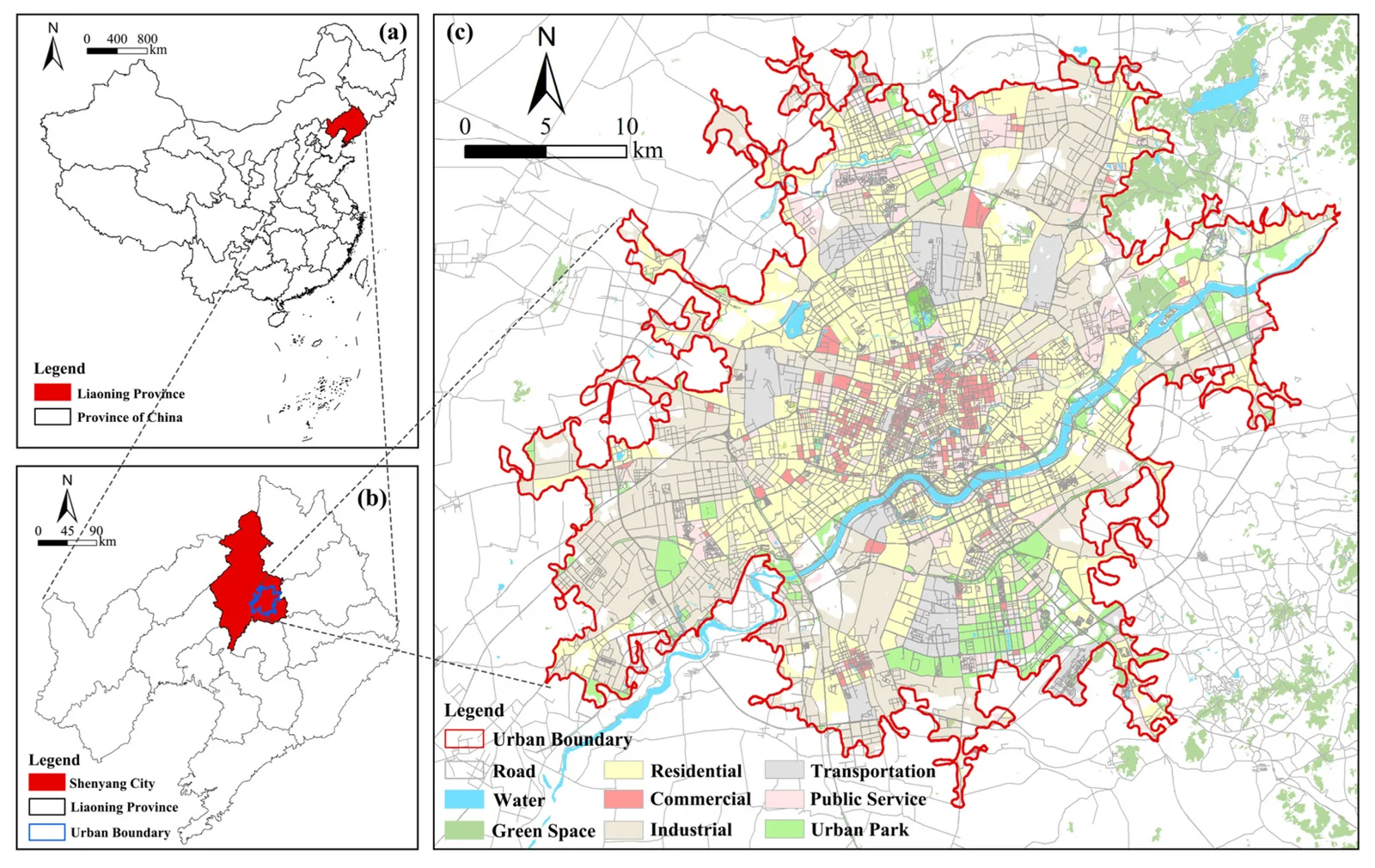
Study area of Shenyang. (**a**) represents Liaoning Province’s location within China; (**b**) represents Shenyang’s location within Liaoning Province; and (**c**) represents the spatial pattern of the study area.

Shenyang (122°25′ E–123°48′ E, 41°11′ N–43°02′ N) is located in central Liaoning Province, China (Figure 4). It has an average elevation of around 50 m and has a temperate semi-humid continental climate [19]. As an important central city in Northeast China, Shenyang has achieved a high level of urbanization, reaching an urbanization rate of 85.5% by 2024 [18]. We selected the main urban area of Shenyang as the study region, with a total area of 1.25 × 10^3^ km^2^. Based on the “Essential Urban Land Use Classification in China” dataset and local conditions, the study region was divided into six classifications: residential, commercial, industrial, transportation, public services, and urban park [20,34].

### 4.2. Data

The data employed in this study included NDVI, land surface temperature (LST), glimmer imager of urbanization (GIU), albedo, land use, essential urban land use classification (EULUC), and urban boundaries (Table 1). Specifically, Landsat 8 data at 30 m resolution and 16-day synthetic were employed to calculate NDVI and albedo data, which were subsequently used to develop UVP data and characterize solar radiation, respectively. The 30 m resolution LST data were utilized to characterize urban thermal environment. GIU data at 10 m resolution and 11-day synthetic were adopted to characterize nighttime light intensity, which effectively captures its fine-scale spatial characteristics within urban areas [35]. Farmland within the urban boundary and outer buffer was excluded using the China Land Cover Dataset (CLCD). Nighttime light duration data were developed by integrating multi-source remote sensing and field observations. The global urban boundary (GUB) data were derived from the Global Artificial Impervious Area (GAIA) product. The urban road data were obtained by downloading from the Open Street Map website. The building height data were derived from published studies and has been verified and utilized by relevant studies [36]. To meet the accuracy requirements of this study, all data were resampled to 30 m before analysis.

### 4.3. Methods

**Figure 5:**
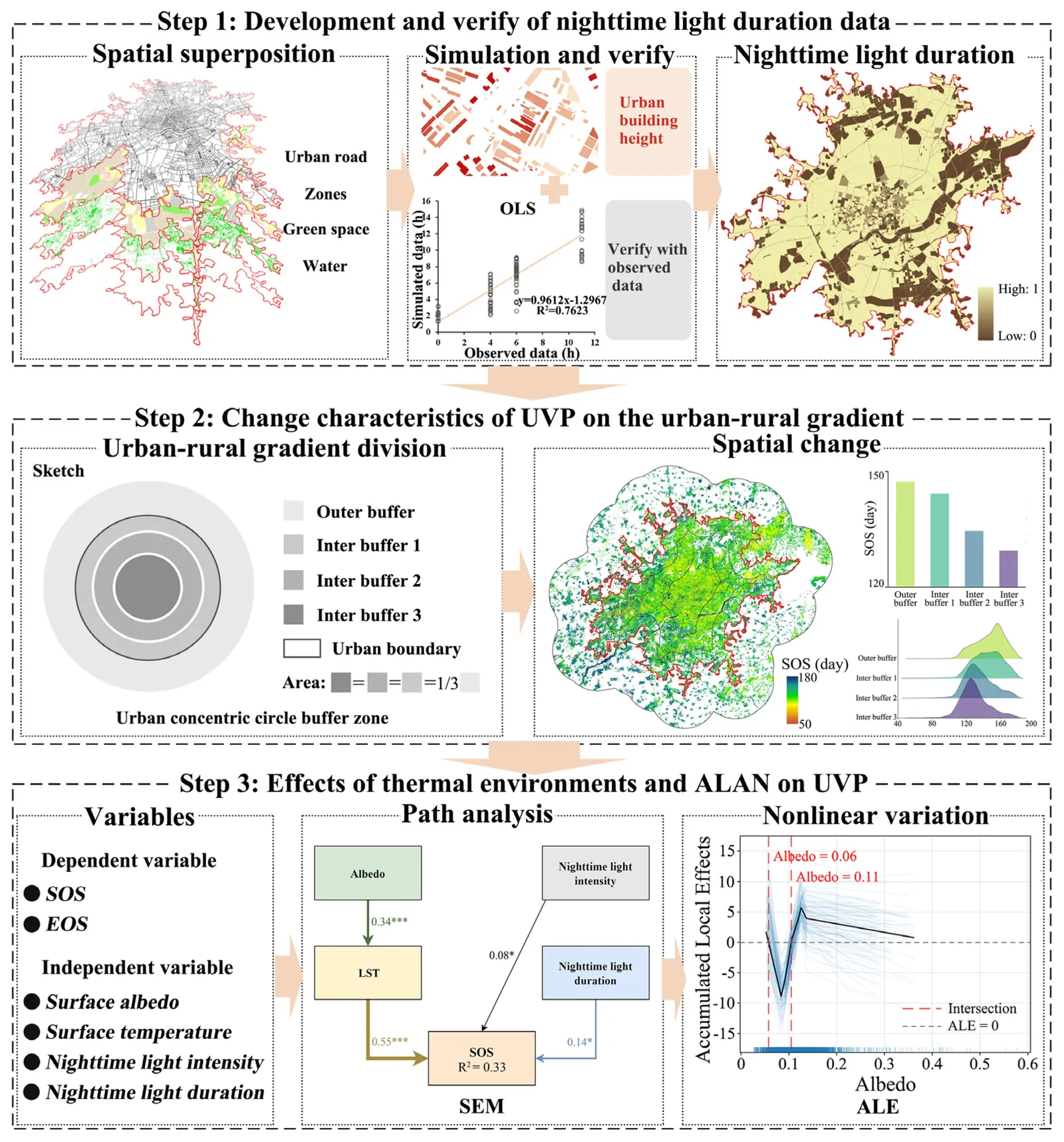
Research framework diagram. The numerals above the arrows represent standardized path coefficients (* *p* < 0.05, *** *p* < 0.001), the blue intervals indicate the distribution of 100 ALE curves determined from 100×Monte Carlo samples. and the black solid line indiactes the mean value of the 100 ALE curves.

As shown in Figure 5, we examined the combined effects of thermal environments and ALAN on UVP in three sections. Firstly, we employed multi-source data to generate comprehensive urban functional zoning data through spatial overlay and assigned values for nighttime light duration to different urban functional zones based on relevant research. The accuracy of the simulated results was verified using field observation data; with a validation R^2^ of 76.2%, the overall accuracy met the requirements. Secondly, we employed the concentric circle buffer method to distinguish between urban areas (inter buffer 1, inter buffer 2, and inter buffer 3) and rural areas (outer buffer), thereby assessing the change characteristics of UVP along the urban–rural gradient. Finally, SEM was applied to identify the pathways and path effects of thermal environments and ALAN on UVP. To further investigate the response characteristics of UVP, the ALE approach was employed to explore the nonlinear influence of drivers on UVP.

#### 4.3.1. Determination of Nighttime Light Duration

We spatially overlapped EULUC, urban road, green space, and water system data to generate comprehensive urban functional zoning data (Figure 5). Due to the complexity of human activities and the diffuse reflection characteristics of light, it is difficult to precisely quantify the nighttime light duration in urban areas [12]. Reference to relevant studies has revealed that there are significant differences in the nighttime light duration across different urban functional zones [6]. We have designated 6:00 PM as the sunset time and assigned nighttime light duration values based on typical lighting patterns across different urban functional zones: residential (6 h), commercial (4 h), industrial (6 h), transportation (11 h), public services (0 h), and urban parks (4 h). To verify the accuracy of the simulation results, we established 15 field measurement sites along the urban–rural gradient, with the sites covering the full range of urban land use types. An automatic continuous-recording illuminance meter was used to record lighting duration. Sixteen field observations were conducted during different seasons in 2023, and the results were compared with simulated data for validation. The validation results yielded an R^2^ value of 76.2%, with the overall accuracy meeting the requirements.

#### 4.3.2. Division of Urban–Rural Gradient

The concentric circle buffer approach was adopted to divide the urban–rural gradient of Shenyang area (Figure 5). To compare the impact of urbanization on phenology, three concentric circular buffers of equal area were delineated inward from the urban boundary [37]. At the same time, to compare the changes in phenological changes between urban and rural regions, concentric circular buffers of equal area to the urban area were delineated outward from the urban boundary. The changes in UVP across the concentric circular buffers (outer buffer, inter buffer 1, inter buffer 2, and inter buffer 3) were used to characterize its spatial pattern along the urban–rural gradient. Furthermore, this classification method has been compared with other urban–rural classification methods in existing studies and has been demonstrated to effectively characterize variations in UVP along the urban–rural gradient [7].

#### 4.3.3. Remote Sensing Inversion of UVP

This study used Landsat NDVI to develop UVP data for Shenyang City in 2023. Compared to commonly used MODIS NDVI data, 30 m resolution NDVI data can more accurately depict the changing characteristics of UVP along urban–rural gradients [38]. To mitigate the influence of satellite sensor features, the double logistic curve was employed to fit the NDVI time series data [39]. This approach effectively reduces the impact of noise in NDVI time series and improves the fitting accuracy of phenological indicators [23]. The specific formula is as follows:

(1) $$g\left(t,a_{1},a_{2},a_{3},a_{4}\right)=\frac{1}{1+\exp\left(\frac{a_{1}-t}{a_{2}}\right)}-\frac{1}{1+\exp\left(\frac{a_{3}-t}{a_{4}}\right)}$$

where *t* is time; *a*_1_ and *a*_3_ control the peaks and troughs of the fitted curve; *a*_2_ and *a*_4_ determine its width and steepness. After fitting the NDVI time series data, key phenological indicators were extracted using the dynamic threshold method. Referring to the previous studies on the Shenyang area, the dynamic threshold of 20% was employed to determine the SOS and EOS [7,40]. In addition, to reduce the influence of outliers, the grids with SOS earlier than the 50th day or later than the 180th day, and those with EOS earlier than the 240th day or later than the 330th day, were removed. In addition, phenological data were compared with the Greenup and Dormancy indices of MCD12Q2 products, yielding R^2^ values of 0.72 and 0.69, respectively, which meet the requirements for precision applications [40,41].

#### 4.3.4. Structural Equation Modeling

This study employed SEM to quantitatively analyze the direct or indirect impacts of thermal environments and ALAN on UVP. As this study was conducted on a medium scale, four factors with relatively high data resolution, namely albedo, LST, nighttime light intensity and duration, were selected based on relevant research [8]. Based on the prior knowledge regarding the impact of urbanization on UVP, we constructed a conceptual path diagram, and the model coefficients were subsequently determined by the maximum likelihood method [5]. It was further hypothesized that albedo, nighttime light intensity, and duration may directly affect the growth processes of urban vegetation while also indirectly affecting it by altering LST. Standardized path coefficients (β) were employed to indicate the extent to which drivers influence UVP, while the coefficient of determination (R^2^) was employed to assess the fitting accuracy of the model [15]. The positive coefficient indicates a positive effect between variables, while the negative coefficient signifies a negative effect. The path effect of the independent variable is quantified by the product of the standardized path coefficients on each path. The following metrics were used to assess the fit of the constructed SEM: chi-square statistic (x^2^)/degrees of freedom (df) < 2.00, comparative fit index (CFI) ≥ 0.90, normed fit index (NFI) ≥ 0.90, incremental fit index (IFI) ≥ 0.90, and root mean square error of approximation (RMSEA) ≤ 0.08. This study selected 2000 sample points using a random sampling method, and after preprocessing and screening, 1669 effective samples were obtained for SEM analysis. The SEM was accomplished using the AMOS (v23) software.

#### 4.3.5. Identifying the Nonlinear Changes in Drivers

After the path analysis of UVP, the ALE model was employed to assess the nonlinear changes in drivers. ALE is a global interpretive method that captures the overall contribution of influencing factors on target factors [42]. The method is not limited by collinearity among influencing factors and can effectively identify both the linear and nonlinear effects of the influencing factors on the target factors [43]. This model is a faster and more efficient alternative to the local dependency plot and has been verified and utilized in related research [44]. We applied the ALE model to investigate the nonlinear relationships between drivers such as thermal environments and ALAN, and UVP. The formula is as follows:

(2) $$f_{1,\mathrm{ALE}}(x_{1})=\int_{x_{\min,1}}^{x_{1}}E\left[f^{1}(X_{1},X_{2})\left|X_{1}=z_{1}\right.\right]{\mathrm{dz}}_{1}-\mathrm{constant}\;\int_{x_{\min,1}}^{x_{1}}\int P_{\left.2\right|1}(\left.x_{2}\right|z_{1})f^{1}(\left.z_{1}\right|x_{2}){{\mathrm{dx}}_{2}\mathrm{dz}}_{1}-\mathrm{constant}$$

where *f*^1^(*x*_1_,*x*_2_)=∂(*x*_1_,*x*_2_)/∂*x*_1_ represents the partial effect of *x*_1_ on the training model *f*(*x*_1_,*x*_2_); *x_min_*_,1_ represents the lower limit value within the selected effective range of *x*_1_; and $\int P_{\left.2\right|1}(\left.x_{2}\right|z_{1})$ represents the conditional status of *x*_2_ when *x*_1_ = *z*_1_.

## 5. Conclusions

Investigating the combined effects of thermal environments and ALAN on UVP is crucial for understanding how human activities influence ecosystem dynamics. This study employed the concentric buffer zone approach to measure the changing characteristics of UVP along the urban–rural gradient in Shenyang, comparing differences between inter and outer buffers. SEM and ALE were employed to identify the combined effects and change thresholds of thermal environments and ALAN on UVP. First, SOS and EOS exhibited gradually decreasing and gradually increasing trends along the urban–rural gradient, respectively. SOS occurred 11.28 days earlier within the city boundary than in the outer buffer, while EOS occurred 3.91 days later. Second, among the EOS path effects, LST (β = 0.41, *p* < 0.05) was the most significant driver, followed by nighttime light duration (β = 0.27, *p* < 0.05) and nighttime light intensity (β = 0.05). The path effects of drivers exhibited pronounced seasonal variations, with the impact of nighttime light duration on EOS (β = 0.27, *p* < 0.05) being significantly greater than its effect on SOS (β = 0.14, *p* < 0.05). Finally, there exists a complex nonlinear relationship between the driving factors and UVP, and the same driving factor exhibits distinct variation patterns with respect to SOS and EOS. The effects of nighttime light duration on SOS and EOS exhibited distinct monotonic decreasing and increasing trends, respectively. Our findings demonstrate that it is crucial to consider both thermal environments and ALAN when assessing the effect of urbanization on vegetation dynamics, particularly for the profound but often overlooked effects of nighttime light duration.

## Figures and Tables

**Table 1:** Data sources of this study.

Variable	Data Product	Resolution	Time	Source
NDVI	USGS Landsat 8 Level 2, Collection 2, Tier 1	30 m/16 day	2023	https://science.nasa.gov/ (accessed on 10 April 2025)
LST	Landsat Collection 2 Surface Temperature	30 m/16 day	2023	https://earthexplorer.usgs.gov/ (accessed on 10 April 2025)
GIU	SDGSAT-1 data	10 m/11 days	2023	https://www.sdgsat.ac.cn/ (accessed on 20 April 2025)
Albedo	USGS Landsat 8 Level 2, Collection 2, Tier 1	30 m/16 day	2023	https://earthexplorer.usgs.gov/ (accessed on 20 April 2025)
Land use	CLCD	30 m/year	2020	https://zenodo.org/records/5816591 (accessed on 20 April 2025)
EULUC	EULUC-China	-	2018	https://data-starcloud.pcl.ac.cn/ (accessed on 20 April 2025)
Urban boundary	GUB	-	2020	https://data-starcloud.pcl.ac.cn/ (accessed on 20 April 2025)
Urban roads	Open Street Map	-	2024	https://www.openstreetmap.org/ (accessed on 20 April 2025)
Building height	Global three-dimensional building footprint dataset	-	2020	https://doi.org/10.5281/zenodo.11397015 (accessed on 20 April 2025)

## Data Availability

The original contributions presented in this study are included in the article.

## Abbreviations

The following abbreviations are used in this manuscript:

ALAN	Artificial light at night
UVP	Urban vegetation phenology
SOS	Start of the growing season
EOS	End of the growing season
SEM	Structural equation model
ALE	Accumulated local effects
LST	Land surface temperature
GIU	Glimmer imager of urbanization
EULUC	Essential urban land use classification
CLCD	China Land Cover Dataset
GUB	Global urban boundary
GAIA	Global Artificial Impervious Area
df	Degrees of freedom
CFI	Comparative fit index
NFI	Normed fit index
IFI	Incremental fit index
RMSEA	Root mean square error of approximation
